# Red blood cell alloimmunization in blood transfusion-dependent β thalassemia major patients in Sana’a City-Yemen

**DOI:** 10.1038/s41598-024-51561-2

**Published:** 2024-01-10

**Authors:** Mohammed A. W. Almorish, Boshra Al-absi, Ahmed M. E. Elkhalifa, Abdulaziz H. Alhamidi, Mohammad Abdelrahman

**Affiliations:** 1 Hematology Department, Faculty of Medicine and Health Sciences, Sana’a University, Sana’a, Yemen; 2 College of Health Sciences, Saudi Electronic University, Riyadh, Saudi Arabia; 3 Clinical Laboratory Sciences Department, College of Applied Medical Science, King Saud University, Riyadh, Saudi Arabia; 4 Clinical Pathology Department, Military Medical Academy, Alexandria, Egypt

**Keywords:** Immunology, Diseases

## Abstract

The development of erythrocyte alloantibodies complicates transfusion therapy in β thalassemia major patients. These antibodies increase the need for blood and intensify transfusion complications. Data on erythrocyte alloimmunization is scarce in Yemeni thalassemia patients. We studied the frequency of alloimmunization in multitransfused β-thalassemia major patients and investigated risk factors that affect antibody formation. Blood samples were taken from 100 β thalassemia major patients who received multitransfused leukodepleted packed red-blood cells. Antibody screening and identification were performed by indirect antiglobulin test using the gel column technique. All patients were tested for autoantibodies using autocontrol and direct antiglobulin test. No adsorption test was done as no autoantibodies were detected in any patient. In our study of 100 β-thalassemia patients, 50 were male and 50 were female with ages ranging from 1 to 30 years. Alloantibodies were present in 6% of patients, while no autoantibodies were detected. Of the 17 alloantibodies identified, the majority were directed against Kell (41.2%) and Rh (29.4%) blood groups. Alloimmunization was significantly associated with age group and sex (p = 0.013, p = 0.030), respectively in β thalassemia major patients. The development of alloantibodies was not significantly associated with duration, total number of transfusions and splenectomy (P = 0.445, P = 0.125, P = 0.647). No autoantibodies found in patients with β thalassemia major. The study found low rates of erythrocyte alloimmunization in multitransfused β-thalassemia major patients, but significant alloantibodies were produced primarily from Kell and Rh blood groups, suggesting the need for providing phenotypically matched cells for selective antigens to improve transfusion efficiency.

## Introduction

Β-thalassemias are types of hereditary anemias with reduced or absent synthesis of β-globin chains, resulting in heterogeneity^1^. Homozygous beta-thalassemia, also called β thalassemia major, impairs β-globin chain production due to mutations in both beta globin genes, causing ineffective erythropoiesis and severe hypochromic microcytic anemia^2^.

Beta thalassemia major is a common chronic hemolytic anemia in children and adolescents worldwide, with the majority of cases occurring in Mediterranean countries and other regions such as the Middle East, West Africa, India, and Southeast Asia. Approximately 60,000 thalassemic children are born globally each year^3,4^. The exact number of individuals with this condition is unknown due to a lack of patient registers in some countries and early mortality in severe cases^5^.

Yemen, a nation of approximately 24 million inhabitants, is an impoverished country where hereditary hemoglobinopathies pose a significant health concern. Notably, the prevalence of thalassemia in Sana'a City was reported to be 13%, with α-thalassemia and β-thalassemia traits accounting for 8.6% and 4.4%, respectively^6^. To manage beta-thalassemia major, a treatment regimen involving regular blood transfusions, spleen removal, folate supplementation, deferoxamine-induced elimination of excess iron, and stem cell transplantation are recommended^7^. In the short term, regular blood transfusions every 3–4 weeks represent the sole practical means of maintaining Hb levels within the range of 9 to 11.5 g/dl. However, this treatment strategy is associated with several long-term challenges, such as alloimmunization, organ damage due to iron overload, and the transmission of blood-borne diseases^8^.

The appearance of antibodies against erythrocytes, whether they are alloantibodies or autoantibodies, can greatly affect the health of thalassemia patients and complicate transfusion therapy. These antibodies can hinder the management of transfusions, leading to delays in obtaining compatible blood and potentially causing hemolysis^9^. Although previous studies have reported a lower incidence of erythrocyte autoantibodies, they can still lead to clinical hemolysis and challenges in blood cross-matching. Individuals with these autoantibodies may need more frequent transfusions and may require immunosuppressive drugs, a splenectomy, or other treatments^10,11^.

There has been no prior investigation that specifically examines the frequency of erythrocyte alloantibodies and autoantibodies, as well as identifies the most common alloantibodies in β-thalassemia major in Yemen. The aim is to assist future efforts in implementing improved blood matching for β-thalassemia patients in Yemen who have received multiple transfusions and are at risk of alloimmunization.

## Patients and methods

Across sectional study was conducted over a period from May to December 2022 at the Yemeni Society of Thalassemia and Genetic Blood Disorders, together with the National Blood Transfusion and Research Center (NBTRC), in Sana'a City.

### Patients

One hundred patients afflicted with β-thalassemia major who received regular blood transfusions were encompassed in this study. All of the patients had previously been diagnosed as individuals with β-thalassemia major after undergoing clinical and laboratory examinations at the Yemen Society of Thalassemia and Genetic Blood Disorders. Subsequently, confirmation of the diagnosis was based on standard hemoglobin electrophoresis.

The patients were admitted for the purpose of receiving a blood transfusion. Blood samples were collected either one month after their most recent transfusion or immediately prior to the transfusion. The patients were all administered ABO and Rh (D) compatible, cross-matched, leukoreduced blood, with the intention of maintaining a target Hb level of 9–11.5 g/dl, in accordance with the transfusion policy of the institution. Pertinent information, such as the age, gender, history of splenectomy, and transfusion history (including age at first transfusion, duration, and number of transfusion units), was duly recorded.

### Methods

Blood samples were obtained via collection in EDTA tubes and subsequently subjected to antibody screening and identification analyses, respectively, in adherence to established protocols. Each sample underwent a direct antiglobulin test (DAT) and autocontrol using a polyspecific antihuman globulin reagent (anti-IgG and antiC3d) via gel technique with low ionic strength saline (LISS). The gel cards used were from Bio-Rad Laboratories, DiaMed GmbH, Switzerland, following Lapierre's method^12^. The DAT reactions were graded on a scale ranging from negative to 4+ as specified by the manufacturer. Plasma samples were screened for the presence of clinically significant alloantibodies by the indirect antiglobulin test utilizing commercial three-cell panel, DiaCell I + II + III test cell reagents with (LISS/Coombs) gel cards (Bio-Rad Laboratories, DiaMed GmbH, Switzerland) at low thermal range and 37 °C incubation with ID-BioRad incubator for warm-reacting alloantibodies.

Plasma samples that exhibited a positive result with the ID-Diacell antibody screen underwent antibody identification utilizing an extended commercial 11-cell identification panel (ID-DiaPanel) using LISS/Coombs gel cards. Samples that yielded a negative result were not subjected to any further testing. The presence of alloimmunization among patients was deemed only if antibodies to one or more erythrocyte antigens could be identified, with both autocontrol and DAT screening yielding negative results. The specificity of the antibodies was established through the interpretation of the reaction patterns of the sample against DiaPanel cells, based on the antibody identification tables provided by the manufacturer.

### Data analysis

The data was analyzed using frequencies and percentages, and the chi square test (χ^2^) was employed for comparison. Exact test was used for expected frequencies less than 5. Significance level was set at p < 0.05. SPSS release version 25 (IBM Corp, Armonk, NY, USA) for Microsoft Windows (2022) was used for statistical calculations.

## Results

### Patient characteristics

Our study consisted of a total of 100 patients, comprising 50 males and 50 females. The demographic and clinical characteristics of the patients are illustrated in Table 1, with the ages of the subjects ranging from 1 to 30 years, and the majority of patients being between 1 to 20 years of age. The frequency of ABO blood groups was as follows: O (63%), A (23%), B (11%), and AB (3%). The RhD antigen was negative in 3% of the patients, as described in Table 1.

**Table 1:** Demographic and clinical characteristics of β thalassemia major patients.

Characteristics	No. of patients (100)	
	No	%
Sex		
Male	50	50
Female	50	50
Age (years)		
1–10	50	50
11–20	45	45
> 20	5	5
Age at start of transfusion		
< 1 year	42	42
1–10 years	53	53
> 10 years	5	5
Spleen state		
Splenectomized	11	11
Non splenectomized	89	89
ABO blood groups		
A	23	23
B	11	11
O	63	63
AB	3	3
Rh typing		
Positive	97	97
Negative	3	3

### Prevalence and identification of alloantibodies

According to Table 2, patients with O positive and A positive blood groups had the highest frequency of alloimmunization, while patients with other blood groups had the lowest frequency.

**Table 2:** ABO blood groups and RH type distribution in β thalassemia major patients.

Blood group &Rh	Alloantibodies		Total
	Negative	Positive	
A+	21	2	23
B+	9	1	10
B−	1	0	1
AB+	3	0	3
O+	58	2	60
O−	2	1	3
Total	94	6	100

The frequency of alloimmunization was observed to be 6%, i.e. 6 out of 100 patients manifested the production of alloantibodies, all of whom were of the female gender. The number of alloantibodies per patient exhibited a range from one to seven. Specifically, two patients (33.3%) displayed one alloantibody, two patients (33.3%) displayed two alloantibodies, while one patient (16.7%) exhibited four alloantibodies. Only one patient (16.7%) was recorded to have developed 7 alloantibodies.

A total frequency of 17 alloantibodies were identified with 8 specificities. The majority (70.6%) of the antibodies were directed against K and Rh systems. Anti K was the most frequent; in 4 patients (23.5%%), followed by antibodies against Kpa 3 (17.7%), while antibodies of Rh system were anti-E 3 (17.7%), followed by anti-c 2 (11.7%). Anti-Lu^b^ and anti Lu^a^ were detected in 2(11.7%) and 1 (5.9%),respectively. Anti-Jk^a^ and anti M were detected in 1 (5.9%) (Table 3).

**Table 3:** Distribution and specificity of alloantibodies in 6 alloimmunized β thalassemia major patients.

Type of antibody	Frequency	
	No	%
RH		
Anti-c	2	11.7
Anti-E	3	17.7
Kell		
Anti-K	4	23.5
Anti-Kp^a^	3	17.7
Kidd		
Anti-Jk^a^	1	5.9
MNS		
Anti-M	1	5.9
Lutheran		
Anti-Lu^a^	1	5.9
Anti-Lu^b^	2	11.7
Total	17	100

### Association of alloimmunization with patients demographic and clinical data

Table 4 presents a summary of the distinctions between patients characteristics with and without alloimmunization in relation to patient-risk factors. The presence of alloantibodies was found to be significantly associated with the patients' sex and age (p = 0.013, p = 0.030), respectively.

**Table 4:** Frequency of alloantibodies in β thalassemia major patient’s in relation to demographic and clinical data.

Variables	Non-alloimmunized (%)	Alloimmunized (%)	Total (%)	*P* value
Gender				
Male	50	0	50	0.013
Female	44	6	50	
Age				
1–10 years	50	0	50	0.030
11–20 years	40	5	45	
> 20 years	4	1	5	
Age of start transfusion				
< 1 years	42	0	42	0.063
1–10 years	48	5	53	
> 10 years	4	1	5	
No. of transfusion				
< 12 unit/year	82	6	88	0.445
≥ 12 unit/year	12	0	12	
Transfusion duration				
< 160 months	66	4	71	0.124
≥ 160 months	28	2	30	
Splenectomy				
Yes	84	5	89	0.647
No	10	1	11	

The presence of alloantibodies was detected in 5 out of 45 patients aged 11–20 years, as compared to only 1 out of 5 patients aged > 20 years, and none out of 50 patients aged 1–10 years. The development of alloantibodies did not show any significant association with the annually transfused blood unites (P = 0.445) and the transfusion duration in months (P = 0.125). Additionally, there was no statistically significant difference in the incidence of alloimmunization between patients who underwent splenectomy (5/89) and those who did not (1/11) (P = 0.647).

### Ethics approval and consent to participate

This study was approved by the Research Ethics Committee of the Faculty of Medicine and Health Sciences, Sana’a University, Yemen. The study was in Accordance with Helsinki Declaration principles. Written informed consent was obtained from all participants.

## Discussion

Erythrocyte alloimmunization may arise in individuals who have undergone multiple blood transfusions. This occurrence can be explained by the vast diversity of erythrocyte antigens present in the 43 blood group systems, which consist of 345 distinct antigens while 33 antigens still have an unknown genetic basis. The emergence of erythrocyte antibodies can result in undesirable consequences such as hemolytic transfusion reactions, and pose challenges in the identification of compatible blood for transfusion therapy in patients with transfusion-dependent β-Thalassemia major^13,14^.

Yemen is one country of the Eastern Mediterranean Region (EMRO) with no published data on the rates of erythrocyte alloimmunization from transfused dependent β thalassemia major patients. This is the first study that examine the rate of alloimmunization among multitransfused β thalassemia major patients in Yemen. The study found that Yemeni multitransfused β thalassemia major patients had a 6% rate of erythrocytes alloimmunization. A literature review of 17 publications from the EMRO showed that rates ranged from 2.87 to 30% among transfused dependent TM patients^15^. Amen et.al reported the highest rate of alloimmunization in Kuwait, while Sadeghian and colleagues reported the lowest in Iran^16,17^. Recent studies in Egypt found alloimmunization rates of 18% and 9% among transfusion dependent β thalassemia major patients^18,19^. This study exhibited a low rate of alloimmunization in patients who had multiple transfusions, which corresponds with other investigations that demonstrated rates varying from 4.24 to 7.4%^20–23^. A study from Thailand discovered a 33.9% rate of erythrocyte alloimmunization in multitransfused thalassemia patients^24^. In Taiwan, Wang et al. reported a 37% incidence of alloimmunization in thalassemia patients receiving transfusion therapy^25^.

Plausible explanation for this low alloimmunization rate in Sana'a City encompass the phenotyping that was performed at NBTRC for both donors and recipients. This comprehensive phenotyping entails the screening of ABO, RH (D, C, c, E, e) and K antigens. In a previous study, it was noted that matching specific antigens decreased alloimmunization in thalassemia patients^19^.

Our study identified eight specificities of antibodies, with the majority (66.7%) of patients displaying more than one alloantibody, resulting in a total of 17 alloantibodies. The antibodies were directed against the Kell (K and Kpa), Rh (E, c), Lutheran (Lua and Lub), Kidd (Jka), and MNS (M) blood group systems. Most of the antibodies (70.6%) were directed towards K and Rh systems, with anti-K (23.5%) being the most frequent, followed by antibodies of anti-E (17.7%), anti-Kpa (17.7%), and anti-c (11.7%).

A study conducted by El-Beshlawy and colleagues revealed that the majority of patients (92%) had more than one alloantibody, with total number of 86 alloantibodies with 12 specificities. The two most prevalent antibody specificities were anti-Kell and anti-Rh^18^. A recently cohort study reported that a total of 27 alloantibodies with 9 specificities were identified in both sickle cell anemia and thalassemia patients. The most frequent alloantibodies in thalassemia patients were anti-K (47%) followed by anti-E (23.5%)^26^. A similar study conducted in Egypt by Osman et al. discovered that anti-K (37.3%) and anti-E (34.3%) were also the most prevalent alloantibodies in thalassemia patients^27^.

Karimi and colleagues found that anti-Kell (K), anti-Rh (D), and anti-Rh (E) were the most common alloantibodies^22^. Shamsian et al. detected anti-K and anti-D in four patients^28^. Bashawri et al. identified Rh and Kell system alloantibodies as most common in Bahrain^29^. In a study by Haslina et al.^30^, anti-E was the most frequent alloantibody, followed by anti-c, anti-S, anti-N, anti-Jka, and anti-K. Bhatti et al.'s research found that Rh antigens were the primary targets of alloantibodies, but anti-K, anti-Jsb, and anti-Jka were also detected^31^. Alloimmunization management varies among countries, depending on factors such as laboratory capabilities, policies regarding donor and recipient typing, and the availability of recommended erythrocytes. There are various concerns related to preventing alloimmunization and its complications. These include the requirement for extended erythrocyte antigen typing prior to transfusion and the significance of blood group genotyping. Additionally, transfusion protocols must match the patient's clinical and immunization status, and immunosuppressive treatments should be used preventively^32^.

In our study, Alloimmunization was more common in females than males, in agreement with previous studies^29,33,34^. Reports from certain regions showed no link between erythrocyte alloimmunization risk in thalassemia patients and female gender. However, female gender has been identified as a risk factor in β thalassemia major patients from Iran, India and Egypt^17,35,36^. Female gender is a significant independent risk factor for alloimmunization, particularly for those who may become pregnant or give birth in the future. As a result of previous alloimmunization, these patients may also have a higher incidence of hemolytic disease in their newborns^26^.

This study revealed a significant association between the development of alloantibodies and age group with the risk being highest in the age group 11–20 years. A study in Oman found a strong link between age and the risk of erythrocyte alloimmunization in β thalassemia major patients, with the highest risk in the 19–30 age group^37^. Other studies have also identified ages over 12 and over 20 as risk factors for alloimmunization^38,39^. The patient's age is an independent risk factor for alloimmunization, with older patients having a higher risk. This is because young children develop acquired immune tolerance to red cell alloantigens^22^. Previous studies have emphasized this finding^9,37^ but it was not be confirmed by other reports^18,24^.

In the present study, the association between alloantibody development and duration and the number of transfusion was found to be non-significant. There is conflicting evidence regarding whether or not a higher frequency and total number of blood transfusions increases the likelihood of erythrocyte alloimmunization. Several research studies have supported the idea that the risk of erythrocyte alloimmunization is higher in patients who have had more frequent transfusions and a higher total number of units^39–41^, although some studies have not found evidence to support this association^42–44^. The findings of El-Danasoury et al. and Thompson et al. suggest that a higher frequency and longer duration of transfusion may increase the risk of alloimmunization in β thalassemia major patients^12,43^.

The prevalence of alloimmunization did not differ significantly between splenectomized and non-splenectomized patients in this study. The literature presents varied findings on the relationship between splenectomy and alloimmunization risk, with some studies showing no association^22,23,30,37,38^, while others report an association^9,17,45,46^. The heterogeneity of transfusion protocols among different centers should be assessed in a large group of patients with standardized transfusion protocols, as this can explain the variation in the rate of alloimmunization in splenectomized patients. The precise cause of the elevated risk of alloimmunization following spleen removal is unclear, but one theory suggests that changes in the shape of red blood cells may increases immunomodulation^9,47^.

## Conclusion

Our study found low prevalence of erythrocyte alloimmunization in multitransfused β-thalassemia major patients. However, most patients developed clinically significant erythrocyte alloantibodies, particularly from Kell and Rh blood groups. Therefore, phenotypically matched cells should be provided for selective antigens, especially Kell and Rh blood groups, to reduce alloimmunization risk and improve blood transfusion efficiency.

## Data Availability

The datasets used and analyzed during the current study are available from the corresponding author on reasonable request.
